# Monte Carlo and Machine Learning-Based Evaluation of Fe-Enriched Al Alloys for Nuclear Radiation Shielding Applications

**DOI:** 10.3390/ma18112582

**Published:** 2025-05-31

**Authors:** Sevda Saltık, Ozan Kıyıkcı, Türkan Akman, Erdinç Öz, Esra Kavaz Perişanoğlu

**Affiliations:** Department of Physics, Faculty of Science, Ataturk University, 25240 Erzurum, Turkey; ozan.kiyikci@atauni.edu.tr (O.K.); turkan.akman16@ogr.atauni.edu.tr (T.A.); erdinc.oz@atauni.edu.tr (E.Ö.)

**Keywords:** Al-alloy, shielding, Fe-rich, gamma, machine learning, Monte Carlo

## Abstract

This study presents a hybrid computational investigation into the radiation shielding behavior of Fe-enriched Al-based alloys (Al-Fe-Mo-Si-Zr) for potential use in nuclear applications. Four alloy compositions with varying Fe contents (7.21, 6.35, 5.47, and 4.58 wt%) were analyzed using a combination of Monte Carlo simulations, machine learning (ML) predictions based on multilayer perceptrons (MLPs), EpiXS, and SRIM-based charged particle transport modeling. Key photon interaction parameters—including mass attenuation coefficient (MAC), half-value layer (HVL), buildup factors, and effective atomic number (Z_eff_)—were calculated across a wide energy range (0.015–15 MeV). Results showed that the 7.21Fe alloy exhibited a maximum MAC of 12 cm^2^/g at low energies and an HVL of 0.19 cm at 0.02 MeV, indicating improved gamma attenuation with increasing Fe content. The ML model accurately predicted MAC values in agreement with Monte Carlo and XCOM data, validating the applicability of AI-assisted modeling in material evaluation. SRIM calculations demonstrated enhanced charged particle shielding: the projected range of 10 MeV protons decreased from ~55 µm (low Fe) to ~50 µm (high Fe), while alpha particle penetration reduced accordingly. In terms of fast neutron attenuation, the 7.21Fe alloy reached a maximum removal cross-section (Σ_R_) of 0.08164 cm^−1^, showing performance comparable to conventional materials like concrete. Overall, the results confirm that Fe-rich Al-based alloys offer a desirable balance of lightweight design, structural stability, and dual-function radiation shielding, making them strong candidates for next-generation protective systems in high-radiation environments.

## 1. Introduction

Radiation shielding is a fundamental requirement in applications where materials are exposed to ionizing radiation, such as nuclear reactors, aerospace systems, and medical radiation therapy facilities. High-energy radiation, such as gamma rays and neutrons, poses serious risks, including material degradation, structural failure, and safety hazards, if not adequately controlled [1]. Traditional shielding materials, such as lead and concrete, offer sufficient attenuation but are hindered by their high density and limited design flexibility. Therefore, the development of lightweight and high-performance radiation-resistant alloys with superior mechanical, thermal, and radiation protection properties has come to the fore [2]. The high gamma radiation absorption capabilities of boridized Fe-Ni alloys have revealed that they are particularly effective in the 662–1332 keV energy ranges [3]. The Nilo 48 alloy, which has a high nickel content, draws attention with its high shielding capacity not only against photons but also against fast neutrons [4]. Studies using Monte Carlo simulations have shown that the Hastelloy C-276 alloy attenuates gamma rays more effectively than many other high-performance alloys [5]. Next-generation high-entropy alloys (HEAs), especially CoCrFeNiSi systems, have surpassed traditional glass and composite materials with their high absorption coefficients despite their low density [6]. Ga-In alloys in liquid metal form can heal micro damages caused by radiation, thanks to their self-repairing structure, and provide the same level of protection as lead with 13% lower weight [7]. In addition, the Bi-10Ag alloy is a strong candidate for medical and nuclear applications, showing high shielding performance against both gamma rays and alpha particles [8]. The gamma-ray attenuation performance of aluminum-based Pb-doped alloys was investigated both experimentally and by simulation. It was found that the radiation shielding capacity increases with increasing Pb content (e.g., 80% Pb), and the PbAl-4 sample has the lowest half-value thickness [9]. Almuqrin et al. [10] calculated the attenuation coefficients of different Fe-, Cr-, and Ni-containing aluminum alloys using the EPICS2017 database and compared them with lead. Some special aluminum alloys were found to be particularly effective against low-energy photons. Al-Fe-Mo-Si-Zr alloys have attracted significant attention due to their lightweight properties, high-temperature stability potential, and sustainable structure [11,12]. These alloys play a critical role in aerospace, nuclear, and structural applications where both performance and longevity are crucial [13,14]. It is known in the literature that the unique combination of elements in Al-Fe-Mo-Si-Zr alloys significantly enhances their mechanical properties and radiation protection capabilities. The addition of Si and Zr plays an important role in improving the microstructure, strength, and thermal stability. Si contributes to the formation of intermetallic compounds such as Al_8_Fe_2_Si, which increase hardness, while Zr inhibits dislocation motion by promoting the precipitation of fine Al_3_Zr dispersions. Such microstructural enhancements result in superior mechanical performance, rendering these alloys ideal for demanding operational conditions [15].

Furthermore, the Mo and Zr elements in these alloys improve radiation shielding properties. Their high atomic numbers contribute to the effective attenuation of ionizing radiation, including gamma rays and neutrons. Studies have shown that alloys containing these elements exhibit superior mass attenuation coefficients, indicating that they are lightweight alternatives to traditional shielding materials [16]. In addition, they are highly durable in radiation-intensive environments, such as nuclear reactors and aerospace applications, due to their corrosion resistance and thermal stability. However, achieving the desired material properties through synthesis poses some challenges, especially in resource-limited laboratory environments. As a result, computational methods have become increasingly important for theoretical validation before experimental application.

Computational modeling plays an important role in evaluating shielding capabilities before synthesis and optimizing material compositions for targeted applications [17]. Theoretical approaches allow a deeper understanding of phase stability, radiation interaction, and mechanical behavior without the limitations of direct synthesis. The integration of computational techniques, such as Monte Carlo simulations, radiation interaction modeling, and machine learning-based predictions, offers a versatile approach to alloy design and performance evaluation [18,19]. These methods provide both low-cost research and insights that can significantly improve alloy development. In this study, a comprehensive computational analysis was used to investigate the fundamental properties of Al-Fe-Mo-Si-Zr alloys. Radiation interaction properties were evaluated using the EpiXS and XCOM databases, which provide precise calculations of mass attenuation coefficients and effective atomic numbers [20,21]. In addition, a Fortran90-based Monte Carlo simulation framework was used to model stochastic radiation transport processes within the alloy system [22,23]. To further improve predictive accuracy, a machine learning-based neural network architecture is trained on multilayer perceptron (MLP) model computational datasets, enabling optimization of alloy compositions and performance predictions [24,25].

This work establishes a hybrid approach to alloy design and characterization by integrating physics-based simulations with predictions. This methodology overcomes the limitations of direct synthesis while providing important insights into material behavior under various conditions. The findings of this research contribute to the broader field of computational materials science by providing a scalable and reproducible framework that can guide future experimental studies and inform industrial applications. The synergy between radiation shielding calculations, Monte Carlo simulations, and machine learning-based predictive modeling offers a new path to design high-performance alloys, ensuring both efficiency and sustainability in next-generation materials engineering.

## 2. Materials and Methods

**Figure 1:**
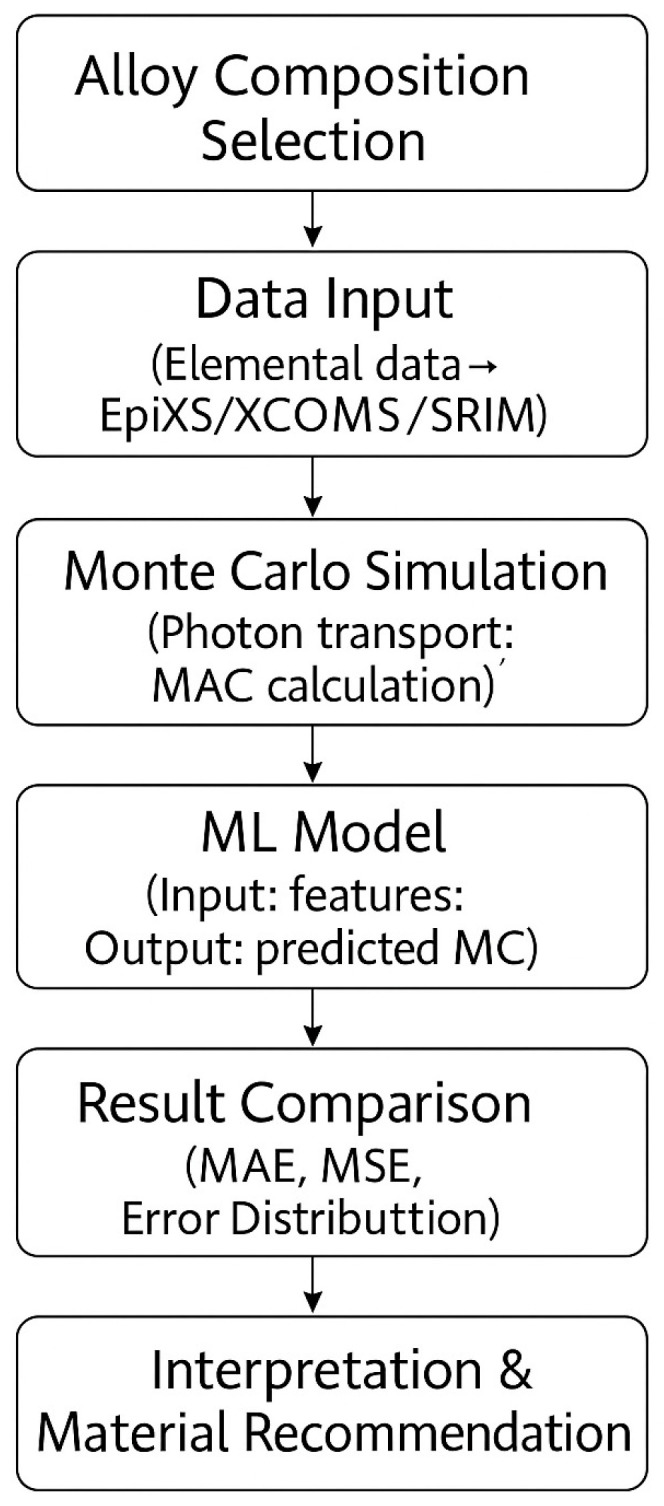
Simulation calculation flowchart for radiation shielding.

The radiation attenuation and shielding capabilities of selected Al-Fe-Mo-Si-Zr alloys [11] were investigated by determining several basic interaction parameters. These calculations were performed using EpiXS (version 2.0.1, Philippine Nuclear Research Institute [PNRI], Quezon City, Philippines) [26] and XCOM (USA, https://physics.nist.gov/PhysRefData/Xcom/html/xcom1.html; accessed on 12 December 2024, National Institute of Standards and Technology [NIST], Gaithersburg, MD, USA) [27] databases that provide theoretical models for photon interactions. The shielding efficiency of the alloys was evaluated by calculating the mass attenuation coefficient (MAC), linear attenuation coefficient (LAC), half-value layer (HVL), and effective atomic number Z_eff_ [28]. The photon interaction probability per unit thickness of the material is defined as the linear attenuation coefficient (LAC) and is calculated using the Lambert-Beer law:

(1) $$I=I_{0}e^{\left\{-\mu x\right\}}$$

where ***I_0_*** and ***I*** are the incident and transmitted photon intensities, respectively, ***µ*** is the linear attenuation coefficient, and ***x*** is the thickness of the material. The mass attenuation coefficient (MAC), which indicates how well a material absorbs photons per unit density, was determined using Monte Carlo simulations and machine learning processes (see Figure 1) that we will discuss in detail in the following steps:

(2) $$\mu_{m}=-\frac{lnT}{\rho x}$$

where ρ is the material density, ***T*** is the fraction of photons transmitted, and ***x*** is the material thickness. These results were validated against theoretical values from the XCOM and EpiXS databases.

(3) $$HVL=\frac{0.693}{\mu }$$

where ***HVL*** represents the material thickness required to reduce the photon density by 50%. The mean free path (***MFP***), which represents the average distance traveled by a photon before interacting with the alloy, was calculated as follows:

(4) $$MFP=\frac{1}{\mu }$$

The effective atomic number ***Z_eff_***, which characterizes the radiation response of a compound, was calculated as follows:

(5) $$Z_{eff}={\left(\frac{\sum f_{i}Z_{i}}{f_{i}A_{i}}\right)}^{\left\{\frac{1}{2}\right\}}$$

where $f_{i}$ is the fractional abundance of element *i*, $Z_{i}$ is the atomic number, and $A_{i}$ is the atomic mass.

To evaluate the neutron shielding capabilities, the fast neutron removal cross-section $\Sigma_{R}$ was calculated for the investigated alloys. The $\Sigma_{R}$ measures the probability of neutron interactions leading to absorption or scattering and is calculated as follows:

(6) $$\Sigma_{R}=\sum_{i}\rho_{i}{\left(\frac{\Sigma_{R}}{\rho }\right)}_{i}$$

where ρ represents the atomic number density of each element in the alloy, and ${\left(\frac{\Sigma_{R}}{\rho }\right)}_{i}$ is the microscopic removal cross-section of element ***i***. The total removal cross-section was determined by summing the contributions of each constituent element. Additionally, the stopping power and projected range of charged particles in the studied alloys were determined using the Stopping and Range of Ions in Matter (SRIM) program (version 2010, Chester, MD, USA) [29]. These theoretical models, machine learning models, and Monte Carlo-based computational results provide a comprehensive understanding of the shielding properties of Al-Fe-Mo-Si-Zr alloys, demonstrating their effectiveness as potential materials for radiation shielding applications.

### 2.1. Monte Carlo Simulation

A Monte Carlo simulation was implemented using Fortran90 (Free Software Foundation, Boston, MA, USA) to compute the mass attenuation coefficients (MACs) of the studied alloys [30,31,32]. This method models radiation transport through the material using stochastic sampling of photon interactions. The simulation process included the following steps:

- Photon scattering interactions.
- Dose absorption and shielding efficiency calculations.

**Figure 2:**
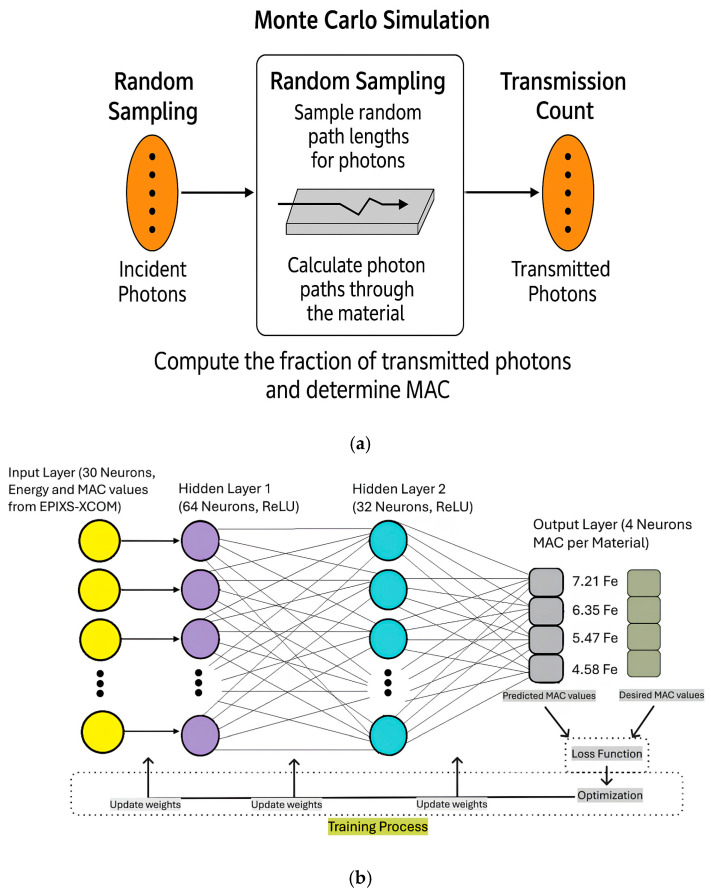
(**a**) MAC calculation using the Monte Carlo method. (**b**) Neural network architecture and training process for MAC prediction.

The Monte Carlo model follows a stepwise random sampling approach to track photon trajectories through the alloy. The probability of interaction is determined using cross-sectional data from nuclear databases. The transport equation is solved iteratively to compute attenuation coefficients (Figure 2a).

The Monte Carlo simulation process in this study follows these steps:

1. Photon Path Length Sampling: Photon path lengths were generated using an exponential probability distribution:
  (7) $$L=-\frac{\ln\left(R\right)}{\mu }$$
  where ***L*** is the photon path length, ***R*** is a uniform random number in (0, 1), and μ is the linear attenuation coefficient.
2. Transmission Calculation: The fraction of transmitted photons through the material was computed as follows:
  (8) $$T=\frac{N_{transmitted}}{N_{total}}$$
  where $N_{transmitted}$ is the number of photons that exit the material, and $N_{total}$ is the total number of incident photons.
3. Mass Attenuation Coefficient Computation: The MAC values were determined by
  (9) $$\mu_{m}=-\frac{lnT}{\rho x}$$
  where ρ is the material density and x is the material thickness. The MAC values were computed across a range of photon energies (keV to MeV) for various alloy compositions, ensuring accurate shielding efficiency assessments. The simulation was started with 10^7^ photon histories to reduce statistical uncertainty. The results were compared with standard XCOM and EpiXS data to validate the theoretical predictions. The Monte Carlo approach provided a detailed evaluation of photon-material interactions, enabling precise assessment of the shielding efficiency of Al-Fe-Mo-Si-Zr alloys.

### 2.2. Machine Learning

In this study, a machine learning approach using multilayer perceptron (MLP) was used to estimate the mass attenuation coefficients (MACs) for different photon energies.

MLPs are among the most common and fundamental components of artificial neural networks. MLPs are widely used to address classification and prediction tasks [33]. These networks are characterized as feedforward networks with at least one hidden layer. Each hidden layer involves multiplying the inputs with certain weights and then passing them through an activation function. The dataset consists of energy-dependent MAC values obtained from experimental and computational sources. MLPs use the supervised learning process. This is the process of trying to reduce the difference between the expected values and the generated values. Information enters the network from the input layer, passes through the hidden layers, and ends up at the output layer, as shown in Figure 2b.

Each input is multiplied by the corresponding weights to form the inputs of the neurons in the first hidden layer. The results of the neurons in the first hidden layer, weighted by synaptic weights, serve as inputs for the neurons in the next hidden layer, and their outputs form the inputs for the output layer.

MLP training is performed in two stages. The backpropagation algorithm (Delta rule) [34] is used for training. The configuration of the neurons can be described using the adopted topology, where each neuron j belongs to a specific layer L. The elements of the weight matrix connecting the *j*th neuron of layer (L) to the *i*th neuron of layer (L-1) are denoted by $W_{ji}^{\left(L\right)}$, and $W_{ji}^{\left(3\right)}$ are the synaptic weights connecting the *j*th neuron of the output layer to the *i*th neuron of the 2nd hidden layer, $W_{ji}^{\left(2\right)}$ are the synaptic weights connecting the *j*th neuron of the 2nd hidden layer to the *i*th neuron of the 1st hidden layer, and $W_{ji}^{\left(1\right)}$ are the synaptic weights connecting the *j*th neuron of the 1st hidden layer to the *i*th neuron of the input layer. The elements of the vectors $I_{j}^{\left(L\right)}$ represent the elements of the weighted (L + 1)th layer of the outputs of the *j*th neuron of the corresponding Lth layer and

(10) $$I_{j}^{\left(1\right)}=\sum_{i=0}^{n}W_{ji}^{\left(1\right)}.x_{i}\Leftrightarrow I_{j}^{\left(1\right)}=W_{1,0}^{\left(1\right)}.x_{0}+W_{1,1}^{\left(1\right)}.x_{1}+...+W_{1,n}^{\left(1\right)}.x_{n}$$

The elements of the vector $Y_{j}^{\left(L\right)}$ are the elements of the corresponding Lth layer and represent the results of the *j*th neuron of the incoming Lth layer and are defined as follows:

(11) $$Y_{j}^{\left(1\right)}=g\left(I_{j}^{\left(1\right)}\right)$$

where ***g*(.)** is an activation operation and is continuous and differentiable.

In the next step, the squared error function indexed by ***k*** is used to compare the desired values with the outputs of the network:

(12) $$E\left(k\right)=\frac{1}{2}\sum_{j=1}^{n_{3}}{\left(d_{j}\left(k\right)-Y_{j}^{\left(3\right)}\left(k\right)\right)}^{2}$$

Here, $Y_{j}^{\left(3\right)}(k)$ represents the value produced by the *j*th result layer neuron for the *k*th data for training, and $d_{j}(k)$ represents the corresponding desired value.

Now we come to the stage of setting the weights for training the network. From the error function, for the *j*th neuron of the final result layer corresponding to the *k*th data for training, we obtain the following:

(13) $$\delta_{j}^{\left(3\right)}=\left(d_{j}-Y_{j}^{\left(3\right)}\right).g^{\prime }\left(I_{j}^{\left(1\right)}\right)$$

where ***g’*(.)** is the 1st-order derivative of the function chosen for activation.

(14) $$W_{ji}^{\left(3\right)}\leftarrow W_{ji}^{\left(3\right)}+\eta .\delta_{j}^{\left(3\right)}.Y_{j}^{\left(2\right)}$$

$\delta_{j}^{\left(3\right)}$ denotes the local gradient associated with the *j*th neuron in the output layer. The weight matrix $W_{ji}^{\left(3\right)}$ is adjusted iteratively, where ***η*** is the learning rate:

(15) $$W_{ji}^{\left(1\right)}\leftarrow W_{ji}^{\left(1\right)}+\eta .\delta_{j}^{\left(1\right)}.x_{i}$$

A similar procedure is performed for the intermediate layers and the input layer to ensure that the network is trained. The intermediate steps are as described by Da Silva et al. [35]. Two fully connected layers with 64 and 32 neurons, respectively, use the ReLU activation functions. To prevent overfitting, early stopping and learning rate reduction techniques were employed. The trained model was used to predict MAC values at specific photon energy levels, comparing results against standard Monte Carlo and XCOM data. The predictive model demonstrated high accuracy, effectively capturing the non-linear dependencies of MAC values on energy levels.

## 3. Results and Discussion

**Figure 3:**
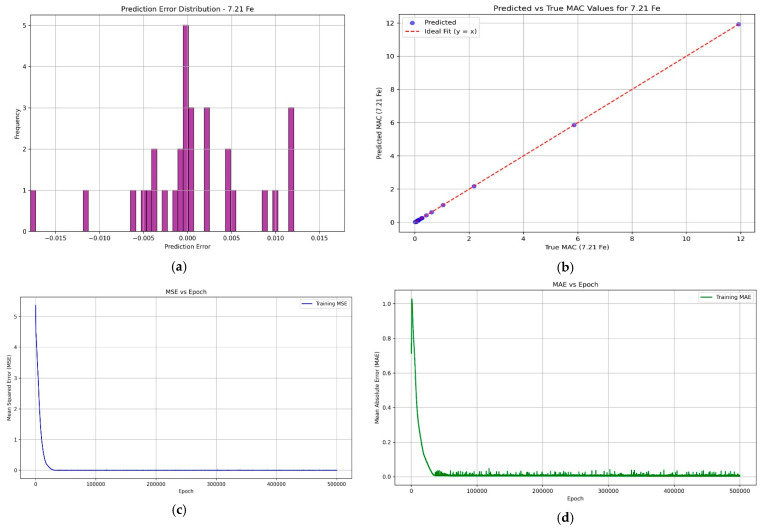
(**a**) Histogram of prediction residuals to assess error distribution, (**b**) scatter plot showing the relationship between predicted and actual MAC values, (**c**) Mean Absolute Error (MAE), and (**d**) Mean Squared Error (MSE) plotted against training epochs.

In this study, the gamma-ray attenuation performance of four Al-based alloys with varying Fe contents (7.21Fe, 6.35Fe, 5.47Fe, and 4.58Fe) was evaluated in terms of the mass attenuation coefficient (MAC, cm^2^/g). These alloys incorporate different proportions of Fe, Mo, Si, and Zr, leading to variations in total heavy metal content, which directly influences photon-matter interaction mechanisms. Figure 3a and Table 1 present a comparison of the MAC values for the 7.21Fe alloy across the photon energy range of 0.015–15 MeV, as calculated using three distinct approaches: machine learning, Monte Carlo Simulation, and EpiXS [26].

In this study, both Monte Carlo (MC) simulations and a machine learning (ML)-based multilayer perceptron (MLP) model were employed to evaluate the radiation shielding properties of Fe-enriched Al-based alloys. While MC simulations provide high-accuracy results based on stochastic physics, they are computationally and memory intensive, requiring scenario-specific modeling for each new composition or energy range. In contrast, the ML model, once trained on validated simulation or experimental data, enables rapid predictions across a broad spectrum of conditions, significantly reducing computation time. This makes ML especially valuable for large-scale material screening, optimization tasks, and real-time shielding evaluations where speed is essential. Additionally, ML models have the capability to extrapolate trends, offering insights into unexplored alloy configurations or energy ranges not explicitly covered in the original dataset (Table 2). The synergy between MC and ML thus presents a powerful framework: MC ensures physical rigor, while ML introduces speed and flexibility together enabling both accurate assessment and efficient exploration of material performance in radiation-intensive environments.

To assess the reliability of the machine learning predictions, four visual analyses were conducted. The MAE and MSE plots over training epochs demonstrate that the model rapidly converges and maintains stability, indicating effective learning and strong generalization ability. The scatter plot of predicted versus true MAC values for the 7.21Fe alloy shows near-perfect alignment with the ideal diagonal, confirming the model’s high accuracy. Furthermore, the residual error distribution exhibits a symmetric, zero-centered spread, suggesting that the errors are random and unbiased. Together, these analyses affirm that the trained model provides robust and accurate predictions, supporting its use as a complementary tool to Monte Carlo simulations for fast and interpretable radiation shielding assessments (Figure 3).

**Figure 4:**
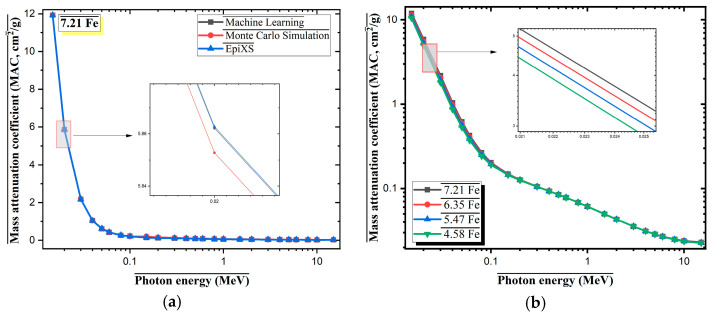
(**a**) MAC values of Al-Fe-Mo-Si-Zr alloy (7.21 wt.% Fe) calculated by ML, Monte Carlo, and EpiXS methods. (**b**) Comparison of MAC values for different Fe contents (7.21, 6.35, 5.47, 4.58 wt.%) using EpiXS.

The results exhibit strong agreement across methods, with minimal deviations observed, particularly in the low-energy region (~0.02 MeV). At this energy level, the MAC reaches approximately 12 cm^2^/g, which corresponds to the regime where the photoelectric effect is dominant [36]. This interaction is strongly dependent on the atomic number (Z), making elements such as Fe (Z = 26), Mo (Z = 42), and Zr (Z = 40) significant contributors to attenuation via photoelectric absorption. In Figure 4, the MAC values of the four alloys with different Fe contents are compared over a broad photon energy range (0.015–10 MeV). In the low-energy region (E < 0.1 MeV), a clear trend is observed, wherein higher Fe content correlates with increased MAC values. This behavior is consistent with the known Z-dependence of the photoelectric effect. For instance, at approximately 0.022 MeV, the 7.21Fe alloy exhibits the highest MAC (~5 cm^2^/g), while the 4.58Fe sample yields the lowest (~3.2 cm^2^/g). In the intermediate energy region (0.1–1 MeV), Compton scattering becomes the predominant interaction mechanism [37]. During this process, photons interact with loosely bound or free electrons, resulting in scattering and energy loss. Since Compton scattering is more dependent on electron density than atomic number, the MAC values of all alloys converge more closely in this range. Both the elemental composition and structural density of the alloys influence this interaction. At higher photon energies (E > 1 MeV), pair production may occur; however, this process becomes significant only in the presence of high-Z elements (Z ≥ 30). In the examined alloys, Zr and Mo may contribute to pair production, though MAC values in this region remain relatively low (~0.05–0.01 cm^2^/g). In conclusion, increasing Fe content enhances the gamma-ray attenuation capability of the Al-based alloys, particularly in the low-energy region where photoelectric absorption dominates. This improvement is attributed to the higher concentration of heavy elements, which intensify photon absorption interactions.

**Figure 5:**
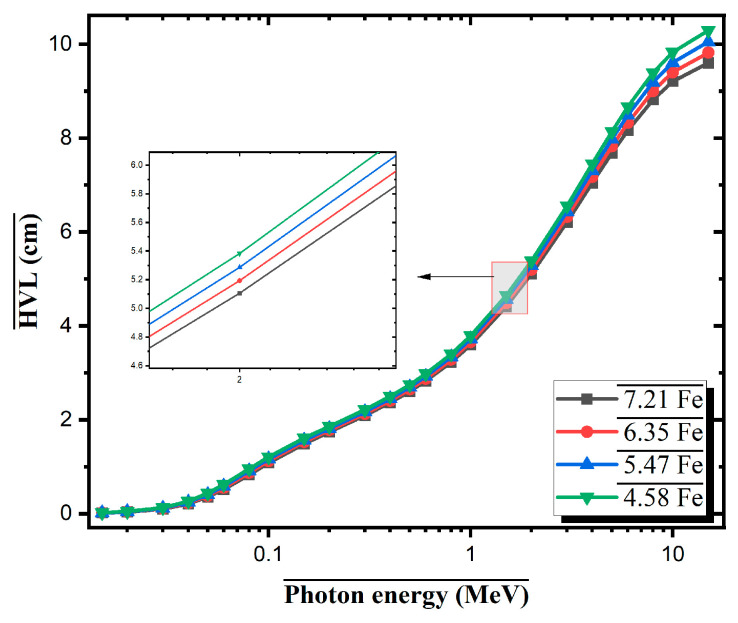
HVL values of Al-Fe-Mo-Si-Zr alloys with varying Fe contents as a function of photon energy.

Figure 5 presents the half-value layer (HVL) values of four Al-based alloys with varying Fe contents (7.21Fe, 6.35Fe, 5.47Fe, and 4.58Fe) over a wide photon energy range from 0.015 to 10 MeV. In the low-energy photon region (E < 0.1 MeV), all alloys exhibit relatively low HVL values, with clearly observable differences among the compositions. For instance, at 0.02 MeV, the 7.21Fe alloy demonstrates the lowest HVL value at approximately 0.19 cm, whereas the 4.58Fe sample exhibits a higher value of about 0.27 cm. This indicates that alloys with higher Fe content exhibit superior attenuation performance against low-energy photons. These findings are consistent with the dominance of the photoelectric effect at low energies, which is significantly influenced by the presence of high atomic number (Z) elements. In this regard, Fe, Mo, and Zr present in the alloy compositions contribute notably to photon attenuation via enhanced photoelectric absorption.

In the intermediate energy range (0.1–1 MeV), Compton scattering becomes the dominant photon interaction mechanism. In this region, HVL values increase gradually, and the differences between the alloy compositions diminish. For example, at 0.5 MeV, the HVL for the 7.21Fe alloy is calculated to be approximately 3.06 cm, while it is 3.24 cm for the 4.58Fe alloy. The reduced disparity in this range can be attributed to the nature of the Compton scattering, which depends more on electron density than atomic number, thereby diminishing the impact of varying Fe content. At higher photon energies (E > 1 MeV), the HVL values increase significantly, reflecting the lower interaction probability of high-energy photons with matter. At 2 MeV, the HVL of the 7.21Fe alloy is approximately 4.85 cm, whereas that of the 4.58Fe alloy is approximately 5.33 cm. As the photon energy continues to increase, the HVL curves for all compositions converge, with values ranging between 9.5 cm and 9.85 cm at 10 MeV. The HVL analysis confirms that increasing the Fe content enhances gamma-ray attenuation performance, particularly in the low-energy regime. The 7.21Fe alloy consistently exhibits the lowest HVL values across all energy ranges, indicating its superior shielding capability.

**Figure 6:**
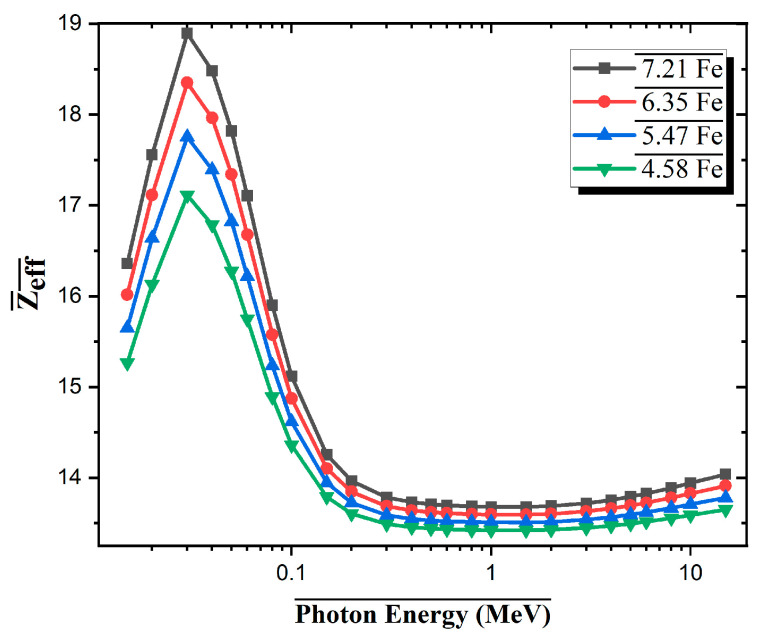
Effective atomic number (Z_eff_) versus photon energy for Al-Fe-Mo-Si-Zr alloys with varying Fe content.

In Figure 6, the effective atomic number (Z_eff_) values of Al-based alloys with four different Fe contents are presented in the photon energy range of 0.015–10 MeV. According to the results obtained, Z_eff_ for all alloys reaches a maximum with a significant peak at low energies, followed by a rapid decrease as the photon energy increases and stabilizes at approximately 13.5–14.5 at high energies. The absorption edges of molybdenum (Mo, K-absorption edge ~0.020 MeV) and zirconium (Zr, K-absorption edge ~0.018 MeV) in the alloy system contribute to the abrupt rise observed at approximately 0.02 MeV. At these energy levels, as the incident photon energy exceeds the inner shell (K-shell) binding energy of the atoms, the probability of photoelectric interaction increases sharply, leading to a sudden jump in Z_eff_ values. For example, the 7.21Fe alloy exhibits the highest effective atomic number in this region at approximately 18.8, while the 4.58Fe sample is approximately 17.0. This difference is directly related to the increase in heavy element content (Fe, Mo, Zr). As the energy increases, Compton scattering becomes dominant, and Zeff values begin to converge for all alloys. The differences in this region are less pronounced because the Compton interaction depends more on the electron density. Among the alloys, the 7.21Fe has the largest Z_eff_ value.

**Figure 7:**
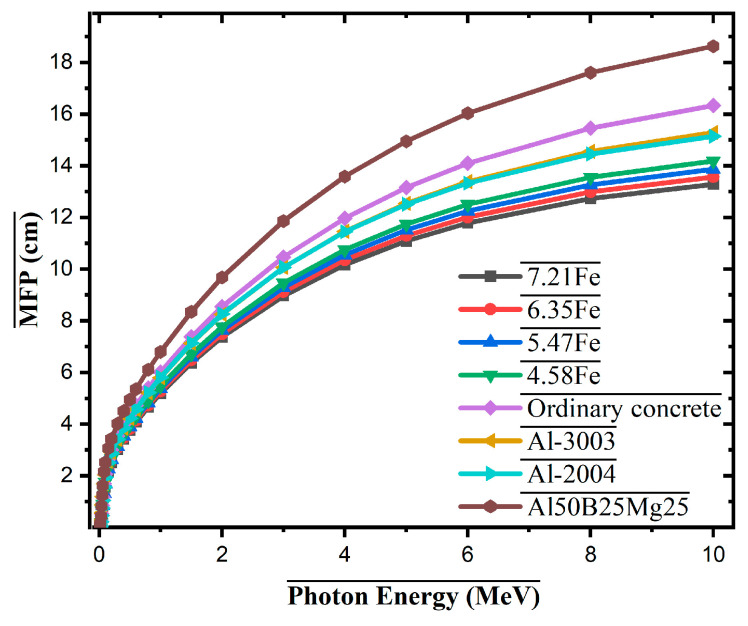
Mean free path (MFP) values of Al-Fe-Mo-Si-Zr alloys compared with conventional materials.

Figure 7 compares the mean free path (MFP) values of the developed Fe-containing Al alloys with selected shielding materials and commercial aluminum alloys over the photon energy range of 0–10 MeV. As expected, MFP increases with photon energy for all materials due to the reduced interaction probability of high-energy photons. Among the tested materials, the 7.21Fe alloy consistently exhibits the lowest MFP values, indicating superior photon attenuation performance. For instance, at 5 MeV, the MFP for 7.21Fe is approximately 11.2 cm, whereas Al50B25Mg25 and ordinary concrete show significantly higher values of approximately 14.8 cm and 13.9 cm, respectively. Compared to commercial aluminum alloys such as Al-3003 and Al-2004, all Fe-based alloys, particularly 7.21Fe and 6.35Fe, demonstrate better shielding efficiency across the entire energy range. This improvement is primarily due to the presence of high-Z elements (Fe, Mo, Zr), which enhance interaction probabilities via photoelectric and Compton processes.

**Figure 8:**
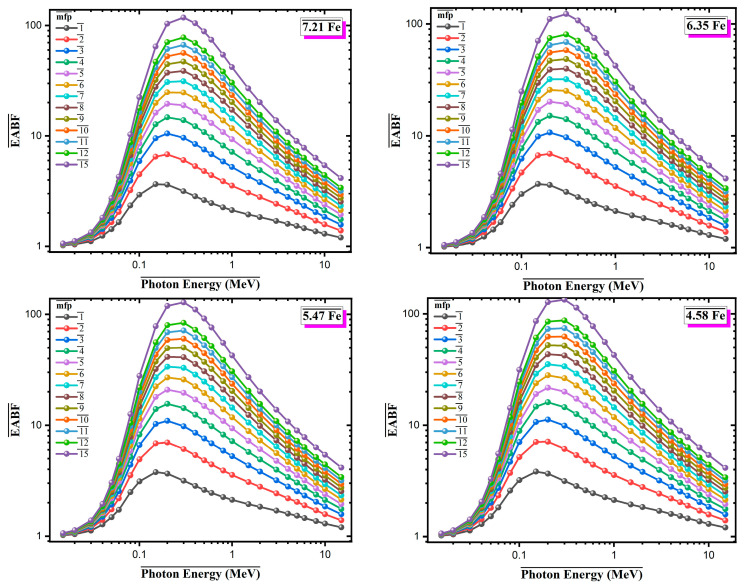
Energy absorption buildup factor (EABF) values for Al-Fe-Mo-Si-Zr alloys with varying Fe contents across different mean free paths (mfp) and photon energies.

**Figure 9:**
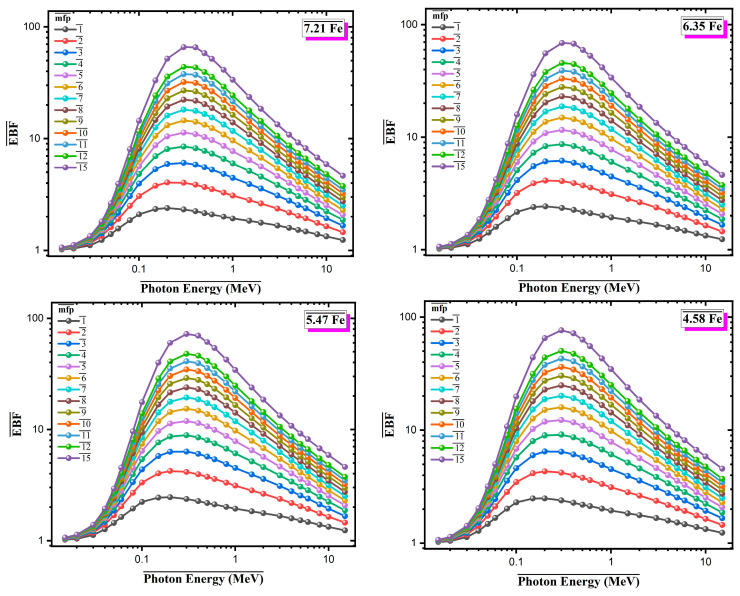
Exposure buildup factor (EBF) values for Al-Fe-Mo-Si-Zr alloys with varying Fe contents across different mean free paths (mfp) and photon energies.

**Figure 10:**
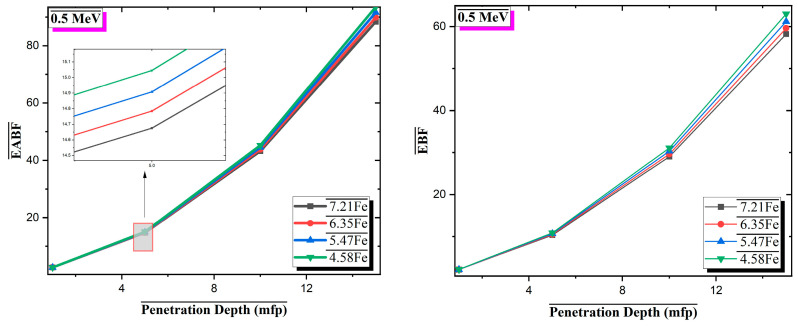
Variation in equivalent (EABF) and exposure build-up factors (EBFs) with penetration depth at 0.5 MeV photon energy for Al-Fe-Mo-Si-Zr alloys.

Figure 8 and Figure 9 present the Energy Absorption Buildup Factor (EABF) and Exposure Buildup Factor (EBF), respectively, for four Al-based alloys with varying Fe contents over the photon energy range of 0.015–10 MeV, at different penetration depths (1–15 mean free paths, mfp). Both EABF and EBF exhibit similar trends across all compositions. The buildup factor values increase in the low-energy region, reach a maximum of approximately 0.4–0.6 MeV, where Compton scattering becomes the dominant interaction mechanism, and then gradually decreases at higher photon energies. In this intermediate energy range, multiple scattering events within the material lead to increased production of secondary photons, contributing to the buildup effect. A clear dependence on Fe content is observed. Alloys with higher Fe content show lower EABF and EBF values across all mfp levels. For instance, at 15 mfp, the 7.21Fe alloy reaches maximum EABF and EBF values of approximately 80–85, whereas the 4.58Fe alloy approaches values close to 100. This reduction in buildup factors is attributed to the presence of high-Z elements (Fe, Mo, Zr), which enhance primary photon attenuation and reduce the number of secondary interactions. At a fixed photon energy of 0.5 MeV, the variation in EABF and EBF with penetration depth was investigated for Al-based alloys with different Fe contents (Figure 10). The results show that both EABF and EBF values increase significantly as the penetration depth increases, due to the cumulative effect of multiple photon scattering and the generation of secondary radiation within the material. Among the four alloys, the 7.21Fe composition consistently exhibits the lowest buildup factors across all depths, while the 4.58Fe alloy shows the highest. For example, at a depth of 15 mean free paths (mfp), the EABF value is approximately 85 for 7.21Fe, compared to nearly 89 for 4.58Fe. This reduction is attributed to the higher content of heavy elements, such as Fe, Mo, and Zr, which enhance photon absorption and reduce energy accumulation. Similarly, EBF values follow the same trend but with less pronounced differences. At 15 mfp, the EBF is about 61 for 7.21Fe and approximately 63 for 4.58Fe. These findings indicate that increasing Fe content in the alloy composition effectively limits the buildup of both energy and exposure, especially at greater depths, thereby improving the overall radiation shielding performance of the material.

**Figure 11:**
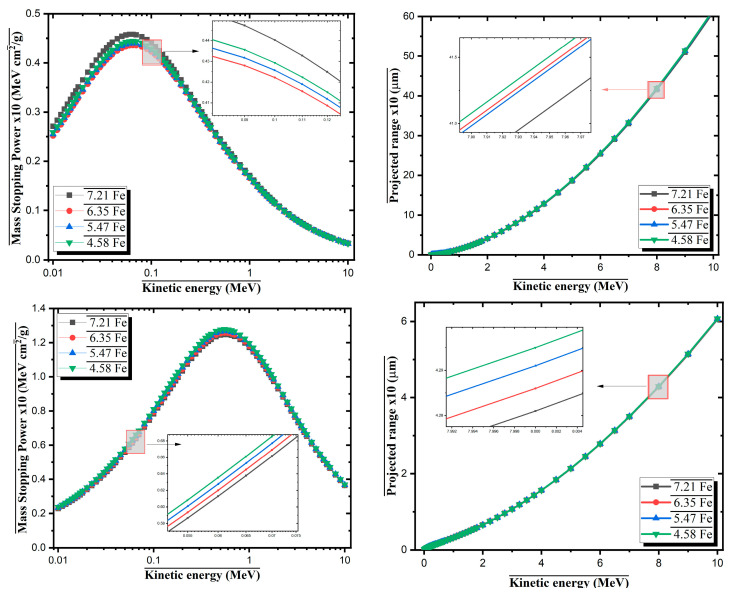
Mass stopping power and projected range for protons and alpha particles in Al-Fe-Mo-Si-Zr alloys.

The interaction behavior of Fe-containing Al-based alloys with charged particles was analyzed using SRIM simulations for both proton and alpha particles. The mass stopping power (MSP) and projected range of the incoming particles were evaluated as functions of kinetic energy for alloys with varying Fe contents (Figure 11). For a proton, the MSP values exhibit a typical Bragg peak behavior, reaching a maximum of approximately 0.1–0.2 MeV for all alloys. Among them, the 7.21Fe alloy shows the highest stopping power (~0.48 MeVcm^2^/g) at the peak, indicating more efficient energy loss due to its higher atomic number and density. As the proton energy increases beyond the peak, the stopping power gradually decreases for all alloys. The projected range of protons increases non-linearly with energy, and at 10 MeV, the 4.58Fe alloy shows the largest penetration depth (~55 µm), while 7.21Fe results in the shortest (~50 µm). This suggests that alloys with higher Fe content are more effective in slowing down and absorbing protons. For alpha particles, a similar Bragg-like profile is observed, with the stopping power peaking at approximately 0.7–0.9 MeV. The maximum stopping power reaches approximately 1.35 MeVcm^2^/g for the 7.21Fe alloy, again confirming the stronger interaction of alpha particles with denser Fe-rich materials. As with protons, the projected range of alpha particles increases with energy. At 10 MeV, the 4.58Fe alloy allows alpha particles to penetrate up to ~6.8 µm, while 7.21Fe restricts it to ~6.2 µm.

**Figure 12:**
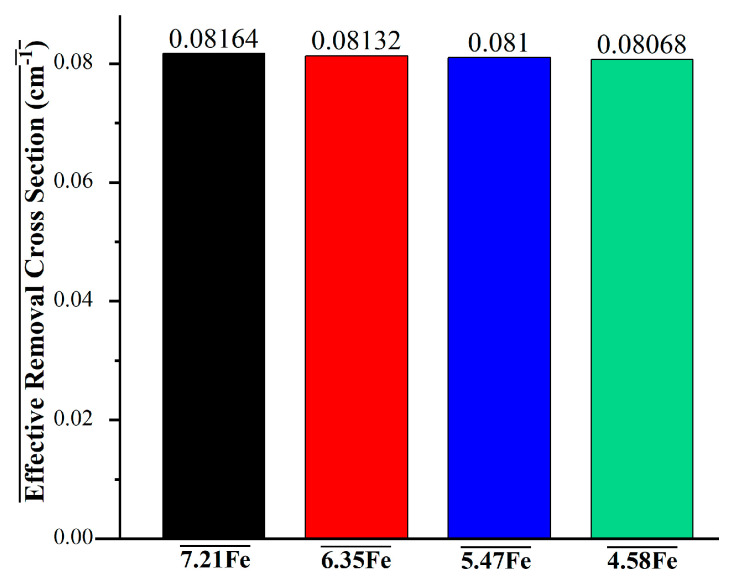
Effective removal cross-section (Σ_R_) values for Al alloys.

The effective removal cross-section (Σ_R_) is a key parameter for evaluating a material’s ability to attenuate fast neutrons through scattering and absorption processes. Figure 12 presents the Σ_R_ values for Al-based alloys with varying Fe content. As shown, all alloys exhibit Σ_R_ values in a narrow range, between 0.08068 cm^−1^ and 0.08164 cm^−1^, indicating similar fast neutron shielding capacities. However, a slight increase in Σ_R_ is observed with increasing Fe content. The 7.21Fe alloy displays the highest removal cross-section (0.08164 cm^−1^), followed by 6.35Fe (0.08132 cm^−1^), 5.47Fe (0.081 cm^−1^), and 4.58Fe (0.08068 cm^−1^). This subtle improvement can be attributed to the higher presence of Fe atoms, which possess moderate neutron interaction cross-sections, thereby contributing incrementally to neutron moderation and capture. Despite the small variation, the data suggest that enhancing the Fe content in Al-based alloys offers a modest improvement in fast neutron shielding performance.

## 4. Conclusions

This study has presented a comprehensive hybrid computational evaluation of Fe-enriched Al-based alloys (Al-Fe-Mo-Si-Zr) for potential use in advanced nuclear radiation shielding applications. Through a combination of Monte Carlo simulations, machine learning-based predictions, and theoretical models (EpiXS, XCOM, and SRIM), key photon and neutron interaction parameters were investigated for four alloy compositions with varying Fe content. The results clearly demonstrate that increasing Fe content enhances gamma-ray attenuation, particularly in the low-energy regime dominated by the photoelectric effect. The 7.21Fe alloy exhibited the highest mass attenuation coefficient (MAC) and the lowest half-value layer (HVL) values, indicating superior shielding efficiency compared to lower Fe content counterparts. The effective atomic number (Z_eff_) and mean free path (MFP) values further confirmed the positive impact of Fe, Mo, and Zr on photon interaction probability. Additionally, the buildup factor analysis revealed that higher Fe content reduces secondary radiation accumulation, enhancing deep-shielding performance. SRIM simulations showed that higher Fe content improves charged particle shielding by reducing the projected range and increasing the stopping power for protons and alpha particles. For fast neutrons, the effective removal cross-section (Σ_R_) increased slightly with Fe content, reaching a maximum of 0.08164 cm^−1^ for the 7.21Fe alloy comparable to standard shielding materials.

Importantly, the machine learning model trained on a multilayer perceptron (MLP) architecture showed excellent agreement with Monte Carlo and database-based results, validating its potential for rapid, accurate material property prediction. In conclusion, Fe-rich Al-based alloys present a promising balance of lightweight design, mechanical stability, and dual-function radiation protection. The hybrid computational methodology adopted here provides a scalable and cost-effective pathway for designing next-generation shielding materials and offers a reliable framework for guiding future experimental validations.

## Figures and Tables

**Table 1:** Mass attenuation coefficients for different Al- Alloys generated using various models (Machine Learning (ML), Monte Carlo Simulation (MC), and EpiXS).

Energy (keV)	7.21Fe			6.35Fe			5.47Fe			4.58Fe		
	ML	MC	EpiXS	ML	MC	EpiXS	ML	MC	EpiXS	ML	MC	EpiXS
15	11.926	11.917	11.927	11.454	11.455	11.455	10.969	10.970	10.970	10.480	10.479	10.481
20	5.862	5.853	5.863	5.575	5.575	5.575	5.281	5.270	5.282	4.980	4.984	4.980
30	2.180	2.179	2.181	2.055	2.054	2.056	1.926	1.927	1.927	1.797	1.795	1.797
40	1.046	1.044	1.039	0.982	0.981	0.983	0.924	0.921	0.926	0.859	0.871	0.868
50	0.607	0.615	0.617	0.588	0.586	0.588	0.561	0.557	0.557	0.540	0.526	0.527
60	0.421	0.425	0.425	0.407	0.407	0.408	0.389	0.389	0.390	0.373	0.372	0.372
80	0.260	0.266	0.266	0.250	0.260	0.258	0.241	0.252	0.250	0.232	0.240	0.243
81	0.258	0.259	0.261	0.249	0.256	0.254	0.240	0.247	0.246	0.231	0.237	0.239
100	0.226	0.203	0.204	0.219	0.200	0.200	0.213	0.197	0.196	0.206	0.193	0.192
150	0.140	0.149	0.148	0.141	0.146	0.147	0.141	0.146	0.145	0.141	0.146	0.144
200	0.126	0.128	0.126	0.126	0.125	0.126	0.125	0.125	0.125	0.125	0.126	0.125
276	0.109	0.109	0.109	0.109	0.110	0.109	0.109	0.108	0.109	0.109	0.108	0.109
300	0.105	0.106	0.105	0.105	0.106	0.105	0.105	0.104	0.105	0.105	0.104	0.105
302	0.105	0.106	0.105	0.105	0.106	0.105	0.105	0.105	0.105	0.104	0.105	0.105
352	0.098	0.098	0.098	0.098	0.099	0.098	0.098	0.098	0.098	0.098	0.099	0.098
383	0.095	0.094	0.095	0.095	0.094	0.095	0.095	0.095	0.095	0.095	0.094	0.095
400	0.093	0.093	0.093	0.093	0.092	0.093	0.093	0.092	0.093	0.093	0.092	0.093
500	0.084	0.085	0.084	0.084	0.085	0.084	0.084	0.085	0.084	0.084	0.083	0.084
600	0.078	0.079	0.078	0.078	0.080	0.078	0.078	0.078	0.078	0.078	0.076	0.078
800	0.069	0.068	0.068	0.068	0.068	0.068	0.069	0.068	0.068	0.067	0.068	0.068
1000	0.049	0.061	0.061	0.049	0.060	0.061	0.048	0.062	0.061	0.049	0.061	0.061
1500	0.047	0.049	0.050	0.047	0.050	0.050	0.047	0.050	0.050	0.047	0.051	0.050
2000	0.045	0.044	0.043	0.045	0.043	0.043	0.045	0.044	0.043	0.045	0.043	0.043
3000	0.041	0.036	0.035	0.041	0.034	0.035	0.041	0.035	0.035	0.041	0.035	0.035
4000	0.038	0.032	0.031	0.038	0.031	0.031	0.038	0.031	0.031	0.038	0.031	0.031
5000	0.034	0.028	0.029	0.034	0.028	0.029	0.034	0.028	0.029	0.034	0.029	0.029
6000	0.030	0.027	0.027	0.030	0.027	0.027	0.031	0.027	0.027	0.031	0.027	0.027
8000	0.023	0.025	0.025	0.023	0.025	0.025	0.023	0.025	0.025	0.024	0.025	0.025
10,000	0.016	0.024	0.024	0.016	0.023	0.024	0.016	0.023	0.024	0.016	0.023	0.024
15,000	0.023	0.022	0.023	0.023	0.023	0.023	0.022	0.023	0.023	0.022	0.022	0.023

**Table 2:** MAC comparison between ML and EpiXS for 7.21Fe alloy.

Energy (keV)	ML Predicted MAC (7.21Fe)	EPIXS MAC Results (7.21Fe)	Absolute Error (ML and EPIXS)
112	0.169	0.176	0.007
1234	0.049	0.054	0.005
1750	0.048	0.046	0.002

## Data Availability

The data presented in this study are available on request from the corresponding author. The data are not publicly available due to privacy.
